# Supplementary material for: Premature utilization of MRI in the workup of mild hyperprolactinemia
Source: PLoS One. 2026 Apr 8;21(4):e0346134. doi: 10.1371/journal.pone.0346134 (PMC13061191; doi:10.1371/journal.pone.0346134)
Supplement: Attachment — Submitted filename: PLOS one response to reviewers_Feb 27.docx [file pone.0346134.s003.docx]

Dear Dr. Premaor,

Thank-you to you and the reviewers for your thoughtful and careful review of our paper “Premature utilization of MRI in the workup of mild hyperprolactinemia” (PONE-D-25-64481). The comments were both insightful and helpful and we have done our best to be as responsive as possible to the reviewer’s concerns. Please find our responses to specific comments detailed below in italics. Changes in the revised manuscript are indicated by tracked changes.

We appreciated your interest in our manuscript and look forward to your decision.

Sincerely,

Kirstie Lithgow

On behalf of my co-authors

1. Please ensure that your manuscript meets PLOS ONE's style requirements, including those for file naming. The PLOS ONE style templates can be found at

<https://journals.plos.org/plosone/s/file?id=wjVg/PLOSOne_formatting_sample_main_body.pdf> and

[https://journals.plos.org/plosone/s/file?id=ba62/PLOSOne_formatting_sample_title_authors_affiliations.pdf](https://can01.safelinks.protection.outlook.com/?url=https%3A%2F%2Ftrack.editorialmanager.com%2FCL0%2Fhttps%3A%252F%252Fjournals.plos.org%252Fplosone%252Fs%252Ffile%253Fid%3Dba62%252FPLOSOne_formatting_sample_title_authors_affiliations.pdf%2F1%2F010f019c73f13453-5b1f3cd7-4758-40b3-9259-a12610da8e5a-000000%2Fr15We9P_u-HTXNuvB7ls9UCmAEfU9RSEwjw7tjSLWsg%3D251&data=05%7C02%7Ckirstie.lithgow%40ahs.ca%7C819dc917654c405257e408de6f66f640%7C11930486b47048f291663f0cc3087727%7C0%7C0%7C639070685446275691%7CUnknown%7CTWFpbGZsb3d8eyJFbXB0eU1hcGkiOnRydWUsIlYiOiIwLjAuMDAwMCIsIlAiOiJXaW4zMiIsIkFOIjoiTWFpbCIsIldUIjoyfQ%3D%3D%7C0%7C%7C%7C&sdata=82MM0jRjme4yU%2BhF1mCCzBUG1HsrNo6Rizw0d6nwdVI%3D&reserved=0)

*Thank you, this has been done.*

2. We noted in your submission details that a portion of your manuscript may have been presented or published elsewhere. “Yes-- abstract published in Endocrine Abstracts due to presentation at European Society of Endocrinology meeting in 2024”. Please clarify whether this [conference proceeding or publication] was peer-reviewed and formally published. If this work was previously peer-reviewed and published, in the cover letter please provide the reason that this work does not constitute dual publication and should be included in the current manuscript.

*A preliminary version of the analysis was submitted to the European Society of Endocrinology annual meeting in 2024 and presented as a poster. All accepted abstracts to that meeting are published in the journal Endocrine Abstracts, which functions as a searchable abstract tool/record from the conference. Many abstracts published here go on to be published in full in other journals. The purpose of presenting at this meeting was to share our preliminary analysis (in poster form) and get feedback from experts in the field which contributed to our final manuscript. The full analysis and other details in our manuscript have not been published elsewhere.*

3. We note that you have indicated that there are restrictions to data sharing for this study. PLOS only allows data to be available upon request if there are legal or ethical restrictions on sharing data publicly. For more information on unacceptable data access restrictions, please see [http://journals.plos.org/plosone/s/data-availability#loc-unacceptable-data-access-restrictions](https://can01.safelinks.protection.outlook.com/?url=http%3A%2F%2Ftrack.editorialmanager.com%2FCL0%2Fhttp%3A%252F%252Fjournals.plos.org%252Fplosone%252Fs%252Fdata-availability%2523loc-unacceptable-data-access-restrictions%2F1%2F010f019c73f13453-5b1f3cd7-4758-40b3-9259-a12610da8e5a-000000%2FizYzdOP7SHe6lgZIKOqoZduZ_SNziKlEXeqpmzkbm4o%3D251&data=05%7C02%7Ckirstie.lithgow%40ahs.ca%7C819dc917654c405257e408de6f66f640%7C11930486b47048f291663f0cc3087727%7C0%7C0%7C639070685446288002%7CUnknown%7CTWFpbGZsb3d8eyJFbXB0eU1hcGkiOnRydWUsIlYiOiIwLjAuMDAwMCIsIlAiOiJXaW4zMiIsIkFOIjoiTWFpbCIsIldUIjoyfQ%3D%3D%7C0%7C%7C%7C&sdata=iDAZmC7DGbDbktr0rdimgyzMU2QcUKSbQAqviaT%2FFFU%3D&reserved=0).

Before we proceed with your manuscript, please address the following prompts:

a) If there are ethical or legal restrictions on sharing a de-identified data set, please explain them in detail (e.g., data contain potentially identifying or sensitive patient information, data are owned by a third-party organization, etc.) and who has imposed them (e.g., a Research Ethics Committee or Institutional Review Board, etc.). Please also provide contact information for a data access committee, ethics committee, or other institutional body to which data requests may be sent.

b) If there are no restrictions, please upload the minimal anonymized data set necessary to replicate your study findings to a stable, public repository and provide us with the relevant URLs, DOIs, or accession numbers. For a list of recommended repositories, please see

<https://journals.plos.org/plosone/s/recommended-repositories>. You also have the option of uploading the data as Supporting Information files, but we would recommend depositing data directly to a data repository if possible.

*Thank you, we have uploaded the data as a supporting file.*

We will update your Data Availability statement on your behalf to reflect the information you provide

4. Please amend your authorship list in your manuscript file to include author Jenna Mann.

*Full name is Jennifer Mann, which has been included.*

5. If the reviewer comments include a recommendation to cite specific previously published works, please review and evaluate these publications to determine whether they are relevant and should be cited. There is no requirement to cite these works unless the editor has indicated otherwise. 


[Note: HTML markup is below. Please do not edit.]

Reviewers' comments:

Reviewer's Responses to Questions

**Comments to the Author**

1. Is the manuscript technically sound, and do the data support the conclusions?

The manuscript must describe a technically sound piece of scientific research with data that supports the conclusions. Experiments must have been conducted rigorously, with appropriate controls, replication, and sample sizes. The conclusions must be drawn appropriately based on the data presented.

Reviewer #1: Yes

2. Has the statistical analysis been performed appropriately and rigorously?

Reviewer #1: Yes

3. Have the authors made all data underlying the findings in their manuscript fully available?

The [PLOS Data policy](https://can01.safelinks.protection.outlook.com/?url=http%3A%2F%2Ftrack.editorialmanager.com%2FCL0%2Fhttp%3A%252F%252Fwww.plosone.org%252Fstatic%252Fpolicies.action%2523sharing%2F1%2F010f019c73f13453-5b1f3cd7-4758-40b3-9259-a12610da8e5a-000000%2FQUrBZW050Gh8MBAu2IU4gR8pTgWnRUVPQ9pSr8Mi9K0%3D251&data=05%7C02%7Ckirstie.lithgow%40ahs.ca%7C819dc917654c405257e408de6f66f640%7C11930486b47048f291663f0cc3087727%7C0%7C0%7C639070685446300361%7CUnknown%7CTWFpbGZsb3d8eyJFbXB0eU1hcGkiOnRydWUsIlYiOiIwLjAuMDAwMCIsIlAiOiJXaW4zMiIsIkFOIjoiTWFpbCIsIldUIjoyfQ%3D%3D%7C0%7C%7C%7C&sdata=fcPXWa4%2F%2FRdaj3RnEY7R1JS3%2BuN0OsH9%2BRFegWwK3Ic%3D&reserved=0) requires authors to make all data underlying the findings described in their manuscript fully available without restriction, with rare exception (please refer to the Data Availability Statement in the manuscript PDF file). The data should be provided as part of the manuscript or its supporting information, or deposited to a public repository. For example, in addition to summary statistics, the data points behind means, medians and variance measures should be available. If there are restrictions on publicly sharing data—e.g. participant privacy or use of data from a third party—those must be specified.

Reviewer #1: Yes

4. Is the manuscript presented in an intelligible fashion and written in standard English?

PLOS ONE does not copyedit accepted manuscripts, so the language in submitted articles must be clear, correct, and unambiguous. Any typographical or grammatical errors should be corrected at revision, so please note any specific errors here.

Reviewer #1: Yes

5. Review Comments to the Author

Please use the space provided to explain your answers to the questions above. You may also include additional comments for the author, including concerns about dual publication, research ethics, or publication ethics. (Please upload your review as an attachment if it exceeds 20,000 characters)

Reviewer #1: I congratulate the authors on this rigorous study addressing a critical gap in clinical practice regarding the overuse of neuroimaging techniques in patients with mild hyperprolactinemia.
I find the authors' focus on the lack of biochemical confirmation before proceeding to costly and often misleading MRI scans to be highly relevant. The finding that 56.1% of MRIs were ordered prematurely highlights a significant healthcare burden and the risk of managing clinically insignificant incidentalomas.
Suggestions for Revision
1. The authors correctly state that guidelines require a second measurement to rule out spurious elevations. In the pediatric and adolescent population, we emphasize that prolactin should be measured at least on two separate occasions to avoid misdiagnosis due to stress or pulsatile secretion. I suggest the authors further emphasize that mild elevations (<100 mcg/L) are frequently transient or related to non-pituitary factors.

*Thank you for your thoughtful and detailed review of our manuscript. We agree with further emphasizing this point and have revised the introduction (lines 72- to 81) accordingly.*

2. The Role of PCOS and Medications: The manuscript would benefit from a more detailed discussion on the common etiologies of mild hyperprolactinemia that must be excluded before MRI. Specifically, Polycystic Ovary Syndrome (PCOS) and antipsychotic medications (e.g., risperidone) are leading causes of mild-to-moderate prolactin elevation. Addressing these would strengthen the clinical rationale for why an immediate MRI is often "premature".

*Thank you for this comment. The association between PCOS and hyperprolactinemia is controversial. While an association has been observed, and a clear mechanistic, causative link has not been established. A recent cross sectional study* ***suggested that PCOS patients (n=1429) do not have higher prolactin levels than controls*** *(1.3% vs 3%, p=0.05) (van der Ham et al Front Endocrinol 2023 Oct 3:14:1245106). Due to uncertainty on this topic, we feel patients with hyperprolactinemia and a concurrent diagnosis of PCOS should still undergo workup for potential alternative etiologies. However, we have added a line indicated that PCOS has been associated with hyperprolactinemia in some studies. We have added additional information about antipsychotic use in in lines 76 to 78.*

3. Macroprolactinemia Screening: A common pitfall in hyperprolactinemia workup is neglecting macroprolactinemia, which accounts for a significant portion of "biochemical" hyperprolactinemia cases. I recommend suggesting that polyethylene glycol (PEG) precipitation should be a standard step before neuroimaging for all mild, asymptomatic cases.

*This has been added (lines 81 to 83).*

4. The "Stalk Effect" and Pseudoprolactinomas: For the "premature" group with abnormal findings, it is essential to distinguish between true prolactinomas and pseudoprolactinomas (non-functioning masses causing stalk effect). In cases where prolactin is <100 mcg/L but a mass is present, the elevation is often due to impaired dopaminergic inhibition rather than a prolactin-secreting tumor.

*Unfortunately, we were not able to access consultation notes through our EMR review (many endocrinologists and other subspecialists work in community clinics outside our academic centre and clinical notes are not reported in our hospital EMR, unlike labs and imaging which are centralized). Presumably, many patients would have undergone endocrinology referral and/or further work-up of hyperprolactinemia to determine etiology (stalk effect vs. prolactin hypersecretion vs. other etiology), but we are not able to specifically report on this.*

5. To provide a broader etiological perspective and support the management of mild hyperprolactinemia in younger populations, I recommend the following recent studies be cited in the Discussion section:
For the Discussion on Etiological Spectrum (especially PCOS):"While pituitary tumors are a known cause, recent evidence in younger cohorts suggests that PCOS is a leading etiology of mild hyperprolactinemia, often showing a significant correlation with BMI-SDS. Incorporating this into the primary workup could significantly reduce the number of unnecessary MRIs." (Reference: Kilci F, et al. Hyperprolactinemia in children and adolescents: clinical characteristics and etiological spectrum. Eur J Pediatr. 2025.)

*Please see our response to #2 above. While we fully acknowledge that this study and others have shown an association of PCOS and mild hyperprolactinemia, a causative mechanism has not been established and evidence on this matter is conflicting. Therefore, it is imperative that these patients still undergo workup for other potential etiologies. We have added the suggested citation with another showing conflicting results to demonstrate the equipoise on this matter.*

For the Discussion on Pituitary Masses and Prolactin Thresholds: "In cases where MRI is performed, it is crucial to recognize that masses associated with prolactin levels below 100 ng/mL are frequently non-functioning pituitary adenomas (NFPAs) or craniopharyngiomas rather than true prolactinomas. These findings support the authors' conclusion that mild elevations rarely predict significant prolactin-secreting tumors." (Reference: Kilci F, et al. Etiology, presentation, and outcomes of hyperprolactinemia due to pituitary masses in children and adolescents. Endocrine. 2025.)

*This has been added (lines 95 to 98)*

6. PLOS authors have the option to publish the peer review history of their article ([what does this mean?](https://can01.safelinks.protection.outlook.com/?url=https%3A%2F%2Ftrack.editorialmanager.com%2FCL0%2Fhttps%3A%252F%252Fjournals.plos.org%252Fplosone%252Fs%252Feditorial-and-peer-review-process%2523loc-peer-review-history%2F1%2F010f019c73f13453-5b1f3cd7-4758-40b3-9259-a12610da8e5a-000000%2FWHbaznYnXyDbFRGZqHG718oWBc2-X7h1JH2zz0MMq68%3D251&data=05%7C02%7Ckirstie.lithgow%40ahs.ca%7C819dc917654c405257e408de6f66f640%7C11930486b47048f291663f0cc3087727%7C0%7C0%7C639070685446312893%7CUnknown%7CTWFpbGZsb3d8eyJFbXB0eU1hcGkiOnRydWUsIlYiOiIwLjAuMDAwMCIsIlAiOiJXaW4zMiIsIkFOIjoiTWFpbCIsIldUIjoyfQ%3D%3D%7C0%7C%7C%7C&sdata=CiUUg1RMX%2FWd3oc%2FNdD8DmA%2FY3fdU9Ua2GsW%2F48l2IQ%3D&reserved=0)). If published, this will include your full peer review and any attached files.


If you choose “no”, your identity will remain anonymous but your review may still be made public.


**Do you want your identity to be public for this peer review?** For information about this choice, including consent withdrawal, please see our [Privacy Policy](https://can01.safelinks.protection.outlook.com/?url=https%3A%2F%2Ftrack.editorialmanager.com%2FCL0%2Fhttps%3A%252F%252Fwww.plos.org%252Fprivacy-policy%2F1%2F010f019c73f13453-5b1f3cd7-4758-40b3-9259-a12610da8e5a-000000%2FABJIcMccGwZTtB5FPUdIz52jQoz4OyeoJrsXHwCPcik%3D251&data=05%7C02%7Ckirstie.lithgow%40ahs.ca%7C819dc917654c405257e408de6f66f640%7C11930486b47048f291663f0cc3087727%7C0%7C0%7C639070685446325241%7CUnknown%7CTWFpbGZsb3d8eyJFbXB0eU1hcGkiOnRydWUsIlYiOiIwLjAuMDAwMCIsIlAiOiJXaW4zMiIsIkFOIjoiTWFpbCIsIldUIjoyfQ%3D%3D%7C0%7C%7C%7C&sdata=wAGuycdXXWq3WqqxCz6CBqWwW%2BoF50wGaTj5rT91oTU%3D&reserved=0).

Reviewer #1: No

[NOTE: If reviewer comments were submitted as an attachment file, they will be attached to this email and accessible via the submission site. Please log into your account, locate the manuscript record, and check for the action link "View Attachments". If this link does not appear, there are no attachment files.]

To ensure your figures meet our technical requirements, please review our figure guidelines: <https://journals.plos.org/plosone/s/figures>

You may also use PLOS’s free figure tool, NAAS, to help you prepare publication quality figures: <https://journals.plos.org/plosone/s/figures#loc-tools-for-figure-preparation>.

NAAS will assess whether your figures meet our technical requirements by comparing each figure against our figure specifications.
